# Conflict over fertilization underlies the transient evolution of reinforcement

**DOI:** 10.1371/journal.pbio.3001814

**Published:** 2022-10-13

**Authors:** Catherine A. Rushworth, Alison M. Wardlaw, Jeffrey Ross-Ibarra, Yaniv Brandvain

**Affiliations:** 1 Department of Plant and Microbial Biology, University of Minnesota, St. Paul, Minnesota, United States of America; 2 Department of Evolution and Ecology and Center for Population Biology, University of California, Davis, California, United States of America; 3 Department of Biology, Utah State University, Logan, Utah, United States of America; 4 Canada Revenue Agency—Agence du revenu du Canada, Ottawa, Ontario, Canada; 5 Genome Center, University of California, Davis, California, United States of America; The Australian National University, AUSTRALIA

## Abstract

When two species meet in secondary contact, the production of low fitness hybrids may be prevented by the adaptive evolution of increased prezygotic isolation, a process known as reinforcement. Theoretical challenges to the evolution of reinforcement are generally cast as a coordination problem, i.e., “how can statistical associations between traits and preferences be maintained in the face of recombination?” However, the evolution of reinforcement also poses a potential conflict between mates. For example, the opportunity costs to hybridization may differ between the sexes or species. This is particularly likely for reinforcement based on postmating prezygotic (PMPZ) incompatibilities, as the ability to fertilize both conspecific and heterospecific eggs is beneficial to male gametes, but heterospecific mating may incur a cost for female gametes. We develop a population genetic model of interspecific conflict over reinforcement inspired by “gametophytic factors”, which act as PMPZ barriers among *Zea mays* subspecies. We demonstrate that this conflict results in the transient evolution of reinforcement—after females adaptively evolve to reject gametes lacking a signal common in conspecific gametes, this gamete signal adaptively introgresses into the other population. Ultimately, the male gamete signal fixes in both species, and isolation returns to pre-reinforcement levels. We interpret geographic patterns of isolation among *Z*. *mays* subspecies considering these findings and suggest when and how this conflict can be resolved. Our results suggest that sexual conflict over fertilization may pose an understudied obstacle to the evolution of reinforcement.

## Abstract 2

Selection can favor the prevention of hybridization by a process known as reinforcement. Inspired by the biology of hybridizing corn subspecies, this study presents a model showing that differences in the costs of hybridization to each sex can undermine this process; this sexual conflict over hybridization results in the transient evolution of reinforcement.

## Introduction

*“Once the pollen grain has arrived at the stigma*, *it has made an irreversible move*. *There should be very intense selection for it to get around whatever barriers the female may erect*.*”*—Janzen (1977)

Reproductive interactions present “different evolutionary interests of the two sexes” [1]. This sexual conflict (in the general sense, sensu [2]) stems from sex differences in the fitness consequences of mating. One sex (often the male) generally benefits from increasing their mating opportunities, while the other sex benefits from choosing mates and/or limiting mating—resulting in the evolution of more specific forms of sexual conflict (e.g., harmful mating tactics and mating evasion; [3]). Because mating and fertilization play a key role in mediating gene flow between divergent populations, sexual conflict can impact the process of speciation. Species boundaries may either be strengthened if sexual conflict poses a barrier to gene flow, or weakened if populations evolve mating tactics that can overcome heterospecific reproductive barriers [1,4–6].

The cost of producing low-fitness hybrid offspring can favor the evolution of enhanced reproductive isolation by a process known as reinforcement [7]. Reinforcement is generally modeled as the evolution of enhanced prezygotic isolation via female preference and male trait, or trait matching [8,9]. Such models generally include trade-offs between being attractive to conspecifics and heterospecifics (i.e., species evolve preferences for different trait values), and as such both sexes tend to benefit from assorting with conspecifics and avoiding the production of low-fitness hybrids. As such, most reinforcement theory aims to address the logistical challenge of maintaining a genetic association between trait and preference [10], rather than the strategic challenge posed by misaligned interests between the sexes. This is true even in models of polygyny (i.e., when females have multiple mates) because males who match a given female trait/preference are assumed to be less attractive to females with a different trait/preference [8,11]. However, if the trade-off between conspecific and heterospecific is less severe, missing conspecific mating opportunities can come at a cost for one sex (usually males), while for the other sex (usually females) the cost of producing a low-fitness hybrid often outweighs the marginal benefit of additional matings.

The imbalance between sexes in the opportunity costs of heterospecific mating sets the stage for sexual selection to impact the evolution of reinforcement. For example, Servedio and Bürger [12] showed that females with preferences for traits maladapted to their environment can favor males expressing these maladaptive traits, enabling persistence of such traits and thus inhibiting the evolution of reinforcement. Similarly, because a male preference results in more competition for mates than does indiscriminate mating [11], reinforcement by male mate choice is more constrained than reinforcement by female choice [8]. Likewise, Aubier and colleagues [9] found that male preference for conspecifics only evolved if potential male effort toward courting unpreferred females could be reallocated to preferred females. These differences in models of reinforcement by male and female mate choice suggest a conflict in the evolutionary interests of the sexes during the evolution of reinforcement; i.e., the benefit of siring low-fitness hybrids may exceed the opportunity cost for a male but not for a female, presenting an overall benefit only to males.

The sexual conflict over reinforcement described above is likely particularly severe for reinforcement of postmating prezygotic (PMPZ) isolation, because reproductive effort cannot be reallocated to preferred partners after mating has already occurred (Janzen’s “irreversible move”, above [13]). From the male perspective, gametes transferred to heterospecific females cannot be redirected, so universally compatible alleles in male gametes will be favored. Whereas from the female perspective, alleles that discriminate against heterospecific gametes in favor of conspecific gametes will be favored.

Motivated by the genetic basis of PMPZ isolation between hybridizing subspecies of *Zea mays* [14], we develop a population genetic model to evaluate how this sexual conflict over hybridization can alter the evolution of reinforcement. To clarify this process, we model one locally adapted “reinforcing” population in which an initially rare female-expressed fertilization barrier requires a population-specific male signal expressed in the pollen or sperm for effective fertilization, and another “non-reinforcing” population, which lacks this male signal and is adapted to a different environment. This female barrier can increase in frequency in the reinforcing population, leading to the initial reinforcement of reproductive isolation. However, the male signal then adaptively introgresses across populations. Fixation of the male compatibility allele across the metapopulation renders the benefit of discerning female-expressed alleles neutral, ultimately eroding reinforcement. Notably, we find a similar outcome when two populations have their own unique incompatibilities (i.e., both species are reinforcing but do so by distinct male signals and female barriers), suggesting that this result is attributable to asymmetric costs and benefits experienced by the sexes, and not simply asymmetric cross-compatibility.

## Results

### Biological inspiration

Gametophytic factors—pairs of tightly linked loci expressed in pollen and styles—underlie the PMPZ barrier [15–19]. Counter to the classic reinforcement prediction that reproductive isolation will be highest in areas of sympatry and reduced in areas of allopatry [20], highland teosinte (*Z*. *m*. subsp. *mexicana*) growing in sympatry with domesticated maize landraces (*Z*. *m*. subsp. *mays*) shows elevated PMPZ isolation from allopatric maize populations, but no elevation in PMPZ isolation from sympatric maize [21,22]. Our model is inspired by the function of gametophytic factors and their puzzling biogeographic distribution.

Despite this inspiration, our model is neither specific nor fully faithful to the maize/teosinte system. We refer to the reinforcing population/species as *reinf* and the non-reinforcing population/species as *non-reinf*, which roughly represent *Z*. *m*. subsp. *mexicana* and *Z*. *m*. subsp. *mays*, respectively. Despite being inspired by a hermaphroditic system, we use the terms “male” and “female” to refer to male and female function, or sperm/pollen and female reproductive tract function, respectively.

### Model overview

We deterministically iterate migration, gamete fusion, and selection, between two populations in secondary contact. See S1 Text for full description of this iteration, and https://github.com/carushworth/gameto-theory for the R code.

#### Population structure, migration, and pollination/mating

**Fig 1:**
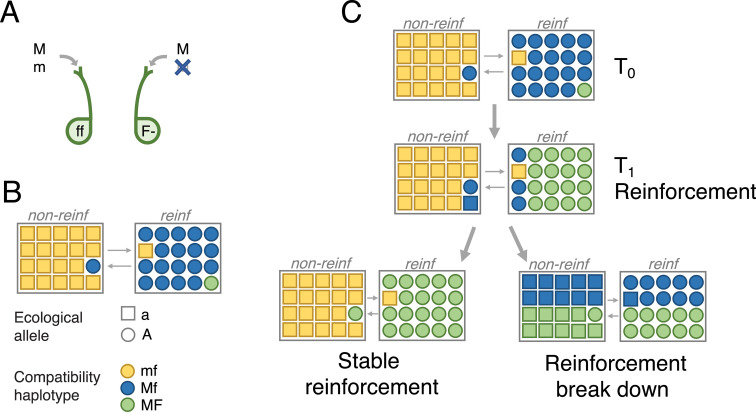
Model dynamics. **(A)** PMPZ incompatibility based on gametophytic factors. The dominant *F* allele at the female-expressed locus F encodes a fertilization barrier that can only be overcome by the male-expressed compatibility allele *M* at locus M. **(B)** Male gametes disperse between two populations: one reinforcing (*reinf*) and one non-reinforcing (*non-reinf*), each of which face different selective pressures. Colors denote compatibility haplotypes (*mf*, *Mf*, *MF*), and shapes signify genotypes at the A locus, which underlies divergent ecological adaptation (*a* and *A*). Initially, *non-reinf* is fixed for the compatible *f* female-expressed allele, the incompatible sperm/pollen-expressed *m* allele, and a locally adaptive allele (haplotype *mfa*). *reinf* is initially fixed for the sperm/pollen compatibility allele *M*, and locally adapted allele *A*, with the female-expressed incompatibility *F* at an initially low frequency; i.e., haplotype *MfA* is initially common, and *MFA* is initially rare in *reinf*. **(C)** We run the model from the initial generation *T*_0_ until the *F* allele reaches its equilibrium. If some reinforcement evolves by time *T*_1_ (equivalent to the *F* allele increasing in frequency in *reinf*), two further outcomes are possible: The *M* allele may introgress onto the locally adaptive background and fix in both populations, leading to the breakdown of reinforcement (bottom right panel of **C**); or, the *M* allele may fail to spread in *non-reinf* while *F* continues to spread through *reinf*, completing reinforcement (bottom left panel of **C**).

We model two demes (i.e., a two-island model) in two differing selective environments. We refer to these as populations or species/subspecies, as the populations represent two locally adapted (sub)species in secondary contact. We refer to the selective environment of each population when appropriate. Every generation, *g*_non-reinf→reinf_ of sperm/pollen in the *reinf* environment originates from the *non-reinf* population. Similarly, *g*_reinf→non-reinf_ sperm/pollen in the *non-reinf* environment originates from the *reinf* population (Fig 1). Within each environment, sperm/pollen and females meet at random.

#### Fertilization

Although mating/pollination within a deme is random, fertilization is controlled by a two-locus PMPZ incompatibility (*sensu* Lorch and Servedio [23], which is a specific form of a “preference/trait” model, with complete female choosiness [24]). The female-expressed locus F is under diploid control. We assume the incompatibility is dominant—i.e., females with one or two *F* alleles discriminate between sperm/pollen alleles, preferentially accepting those with the *M* allele (Fig 1A) at the male compatibility (M) locus. Fertilization is random for females homozygous for the compatibility allele, *f*. This notation differs from that in the existing literature on gametophytic factors, which refer to gametophytic factors as haplotypes rather than pairs of genotypes (see S2 Text).

We initially assume no direct fitness cost to either the female incompatibility *F* (e.g., there is no preference cost) or the male compatibility *M*, unless otherwise noted. Finally, we assume that females expressing the incompatibility genotypes cannot be fertilized by incompatible sperm/pollen. Incomplete penetrance of the barrier results in expected quantitative differences in results but does not change the qualitative outcomes (S2 Fig).

#### Selection

We model isolation by local adaptation following [25] with extrinsic isolation driven by *n* local adaptation loci, each denoted as $A_{i}$, where the subscript *i* is an arbitrary index of loci. Our primary analyses focus on the case of one locally adaptive locus (i.e., *n* = 1), an assumption that is relaxed where noted. Selection coefficients are *s*_non-reinf_ and *s*_reinf_ in environments of the non-reinforcing and reinforcing populations, respectively. Fitness *w* is multiplicative within and among loci, *w* = (1−*s*_env_)^#maladapted alleles^.

#### Initial allele frequencies

At the female-expressed F locus, we assume that the incompatibility allele, F, is initially rare in *reinf* (1% frequency) and absent in *non-reinf*.

At the male-expressed M locus, we assume that the male compatibility allele *M* is initially fixed in *reinf*, and absent in *non-reinf*, respectively. Variation in the initial frequency of *M* in *reinf*, however, has nearly no effect on the outcome (S1 Fig).

At the locally adaptive A locus, we assume that populations are initially fixed for the allele locally adapted to their environment (i.e., allele *A* is fixed in *reinf* and absent in *non-reinf*, and allele *a* is fixed in *non-reinf* and absent in *reinf*).

#### Recombination and genome structure

We initially assume a locus order of $A_{1}MF$, with recombination rates $r_{A_{1}M}$ and $r_{MF}$. Local adaptation loci $A_{2}$ through $A_{n}$ are unlinked to one another and to the M and F loci. After presenting these results, we explore alternative marker orders.

#### A second gametophytic factor

To ensure that our results are not due to asymmetry of variation for female choice in only one population, we then introduce a model with a second unlinked incompatibility locus: $A_{z}M_{z}F_{z}$. This barrier acts like the first, detailed above, but with initial frequencies in each population reversed. As such, each population is reinforcing and non-reinforcing at a different set of loci.

### Sexual conflict leads to transient reinforcement

**Fig 2:**
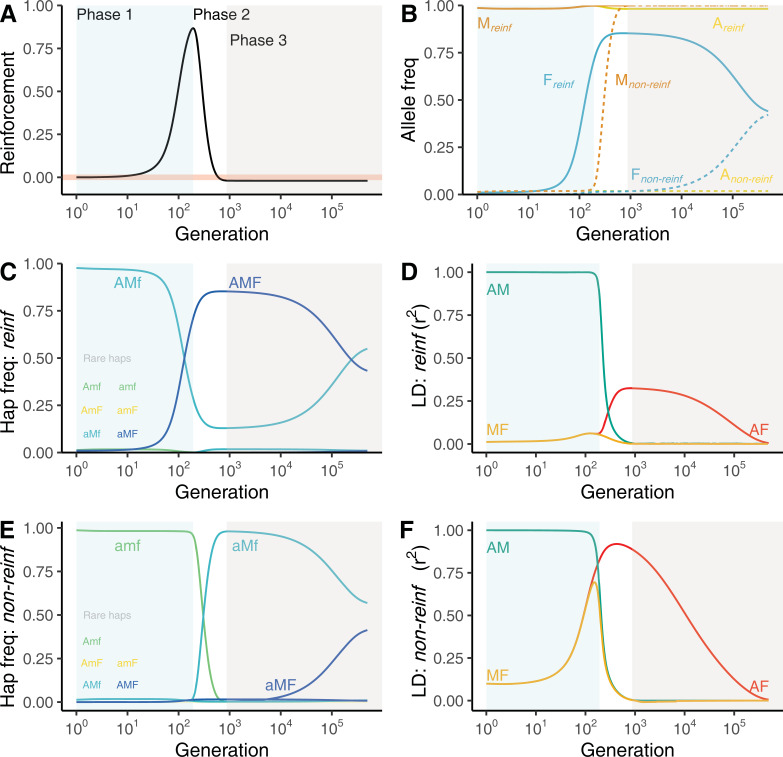
The rise and fall of reinforcement in 3 phases. A female barrier allele *F* preventing fertilization by *m* gametes spreads in the reinforcing population/species *reinf* (Phase 1: light blue). The compatible sperm/pollen allele, *M*, next introgresses into the non-reinforcing population/species *non-reinf* and spreads (Phase 2: white). After *M* fixes, the barrier allele *F* slowly disassociates from the reinforcing background, eventually equilibrating in both populations (Phase 3: light grey). (**A**) Reinforcement is transient, building in Phase 1 and breaking down in Phase 2. The pink line shows 0 reinforcement. (**B**) Allele frequencies in both populations, with solid and dashed lines showing frequencies in *reinf* and *non-reinf*, respectively. The *F* allele increases in *reinf* followed by the global fixation of *M* and subsequent neutrality of *F*. (**C**) Haplotype frequencies and (**D**) gametic linkage disequilibrium (LD) between all pairs of loci over time in the environment of *reinf*. (**E**) Haplotype frequencies and (**F**) gametic LD between all pairs of loci over time in the environment of *non-reinf*. LD is measured as *r*^2^, and all measures describe populations after selection and before recombination. This figure illustrates a single set of parameter values with 1 adaptive locus. Selection: *s*_reinf_ = *s*_non-reinf_ = 0.75; Migration: *g*_non-reinf→reinf_ = *g*_reinf→non-reinf_ = 0.1; Recombination: $r_{AM}=r_{MF}=0.0001$; Initial allele frequencies: *f*_*M*0,reinf_ = 1, *f*_*M*0,non-reinf_ = 0, *f*_*F*0,reinf_ = 0.01, *f*_*F*0,non-reinf_ = 0. The data underlying this figure can be found in https://datadryad.org/stash/dataset/doi:10.5061/dryad.rjdfn2zf8.

When reinforcement evolves, it is almost always transient. An example of the rise and fall of reinforcement is shown in Fig 2 (parameter values in legend). Fig 2A shows that the evolution of substantial reinforcement (Phase 1; Fig 2A) is ultimately fleeting. Reinforcement begins as the female incompatibility allele, *F*, increases in frequency in *reinf* (Fig 2B), preventing fertilization by locally maladapted immigrant haplotypes. This maintains both the high frequency of locally adapted (*A*) and male compatible (*M*) alleles in the environment of *non-reinf* (Fig 2C), and large nonrandom statistical association, a.k.a. linkage disequilibrium (hereafter, LD) between them (note the large value of $r_{AM}^{2}$ in Fig 2D and 2F).

Subsequently, however, the male compatibility allele *M* introgresses into *non-reinf*, eventually recombining onto the *a* background and undermining reinforcement (Phase 2 of Fig 2); i.e., migration of haplotypes from *reinf* into *non-reinf* enables recombination of *M* onto the locally adapted background. As the *Mfa* haplotype sweeps through *non-reinf* (Fig 2E), LD between *M* and *A* decreases in both populations (Fig 2D and 2F).

**Fig 3:**
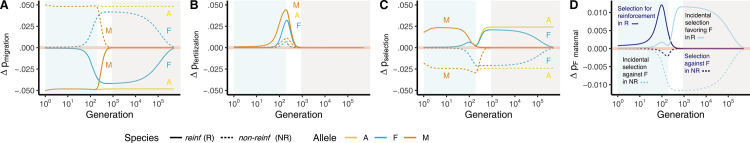
Allele frequency change across the life cycle. The change in frequency of alleles initially unique to *reinf* (*A*, *M*, and *F*) across the metapopulation. The transparent thick pink line at 0 denotes no change in allele frequency during this life phase. (**A**) During migration, alleles decrease in frequency in *reinf* (solid line, indicated by values below the 0 line) and increase in *non-reinf* (dotted line, indicated by values above the 0 line). (**B**) During fertilization, alleles native to *reinf* (*A*, *M*, and *F*) increase in both populations. This effect is strongest in the environment of *reinf*, due to a direct fertilization advantage of *M* and the benefit to alleles in LD with *M*. (**C**) Selection in *reinf* and *non-reinf* consistently acts to increase and decrease the frequency of *A*, respectively. Linkage between *A* and *M* results in overlapping lines during Phase 1. The transition from Phase 1 to Phase 2 is marked by a dip in the frequency of *A*, caused by near-fixation of *F* on the *A* background, as migrant haplotypes from *non-reinf* are unable to penetrate *reinf* at *F*’s peak frequency. (**D**) Selection on *F* is decomposed into 2 components of allele frequency change. In dark blue, we show “selection for reinforcement” (the *F* allele frequency change attributable to preferential fusion with *M*), which enables avoidance of the maladapted a allele in *reinf*. In light blue, we show the allele frequency change attributable to the incidental gametic phase linkage between *F* and *A*; see Materials and methods for more detail. **Parameter values**: One local adaptation locus with Selection: *s*_reinf_ = *s*_non-reinf_ = 0.75; Migration: *g*_non-reinf→reinf_ = *g*_reinf→non-reinf_ = 0.1; Recombination: $r_{AM}=r_{MF}=0.0001$; Initial allele frequencies: *f*_*M*0,reinf_ = 1, *f*_*M*0,non-reinf_ = 0, *f*_*F*0,reinf_ = 0.01, *f*_*F*0,non-reinf_ = 0. Background shading marks Phase 1 (light blue), Phase 2 (white), and Phase 3 (grey) of transient reinforcement, as in Fig 2. The data underlying this figure can be found in https://datadryad.org/stash/dataset/doi:10.5061/dryad.rjdfn2zf8.

As *M* rises in frequency and eventually fixes across the metapopulation (Fig 2B), migrant sperm/pollen are no longer rejected, indicated by reinforcement approaching zero in Fig 2B. At this point, selection against *F* in *non-reinf* weakens until it is completely neutral (see discussion of Fig 3, below). From then on, *F* slowly equilibrates across populations (Phase 3 of Fig 2A) as continued migration and recombination between the A and F loci decreases LD between them (Fig 2D and 2F).

### Allele frequency change across the life cycle

We now show how migration, fertilization, and selection drive changes in allele frequencies across the life cycle (Fig 3). See S3 Text for exact expressions.

#### Migration homogenizes allele frequencies

The change in allele frequency by migration is the difference in allele frequencies between populations weighted by the migration rate (Eq. S1). This homogenization of allele frequencies (Fig 3A) is seen as the decrease in frequency of all “local alleles” (*A*, *M*, and *F* always decrease in *reinf* and increase in *non-reinf*).

#### The stylar barrier *F* favors the male compatibility allele *M* and indirectly favors alleles in LD with it

The fertilization advantage of *M* depends on the proportion of incompatible females in the present generation (either heterozygous or homozygous for *F*). For a dominant female incompatibility, this equals 1−*p*_*ff*_, where *p*_*ff*_, the frequency of females homozygous for the *f* allele, differs from $p_{f}^{2}$ due to nonrandom fertilization. The increase in frequency of allele *M* (derived in Eqs. S2 and S3) from sperm/pollen to paternally derived haplotypes equals

$$\Delta p_{\mathrm{fertilization}}=(1-p_{ff})\frac{p_{M}^{\prime }p_{m}^{\prime }c}{1-cp_{m}^{\prime }}$$ (1)

where *c* is the intensity of incompatibility (or choosiness), and the superscript ′ indicates that allele frequencies in sperm/pollen are taken after migration, while female frequencies lack ′ because only sperm/pollen migrate.

In line with this result, Fig 3B shows that in both populations, the male compatibility allele, *M*, increases in frequency during fertilization until it reaches fixation. In addition to directly increasing the frequency of the *M* allele, selection indirectly favors alleles in LD with it (Eqs. S4 and S5). Because LD among alleles from *reinf* >0, the *A* and *F* alleles increase in frequency through a fertilization advantage to *M* in both populations (Fig 3B). This incompatibility system generates a *trans* association between maternally derived F and paternally derived M alleles (Eq. S6; [26]).

#### Allele frequency change by natural selection follows standard expectations

Selection increases the frequency of the locally adapted allele at locus A in each environment (Fig 3C; Eqs. S7 and S8). Likewise, linked selection on *M* and *F* alleles (Fig 3C) reflects LD with the locally adapted alleles (Fig 2D and 2F), with alleles in positive LD with *A* increasing in frequency in *reinf*, and decreasing in *non-reinf* (Eq. S9).

#### Selection favors the female incompatibility in the reinforcing population and disfavors it in the non-reinforcing population

The female incompatibility allele, *F*, which does not itself impact fitness, still deterministically changes in frequency due to its LD with alleles at other loci. This LD is generated by both the causal effect of the allele in mediating nonrandom fertilization, as well as population structure, historical events, etc. We partitioned the extent to which the increase in frequency of the female isolating barrier *F* is attributable to its causal effect on creating genotypic LD by imposing assortative fertilization (which we call “selection for reinforcement”) versus “incidental selection” unrelated to the effect of *F* on preferential gamete fusion (see Materials and methods for details). “Selection for (or against) reinforcement” reflects the increase (or decrease) in frequency of *F* attributable to the *trans* LD it immediately generates with the locally (mal)adapted allele. “Incidental selection” reflects the change in frequency of *F* attributable to its LD (largely in *cis*) with the locally (mal)adapted allele generated by previous mating and/or historical population structure.

*F* initially rises in frequency in *reinf* because at the outset it preferentially fuses with sperm/pollen unlikely to contain a locally maladapted allele (Phase 1; Fig 3D). However, *F*’s persistence once *M* has reached appreciable frequency in *non-reinf* is primarily attributable to incidental selection, in that it preferentially exists on locally adapted haplotypes (Fig 3D).

In *non-reinf*, *F* is disfavored through both selection against this incompatibility (because *F* is preferentially fertilized by *A*-bearing sperm/pollen) and incidental selection acting on the *A* locus (because *F* is in gametic phase LD with the locally maladapted *A* allele). As recombination erodes LD between *M* and *A*, selection weakens against the incompatibility *F* in *non-reinf* due to its effects on nonrandom fertilization. Incidental selection against *F* in *non-reinf* similarly weakens as recombination erodes LD between *F* and *A* (Fig 3D).

#### Determinants of the strength and duration of reinforcement

**Fig 4:**
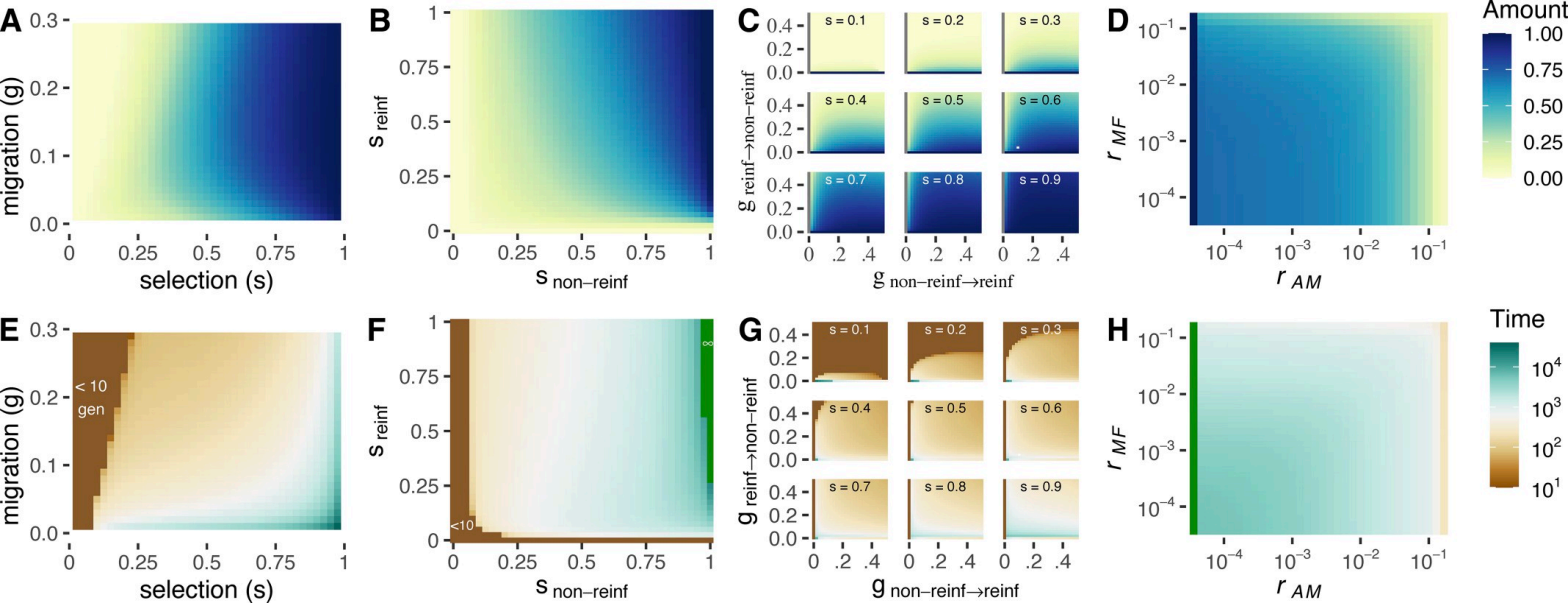
The maximum amount (A–D) and duration (E–H) of reinforcement. Reinforcement as a function of symmetric selection and migration (**A** and **E**) with $r_{AM}=r_{MF}$ = 10^−4^, different selection coefficients in reinforcing and non-reinforcing populations’ environments (**B** and **F**), with *g*_non-reinf→reinf_ = *g*_reinf→non-reinf_ = 0.03 and with $r_{AM}=r_{MF}$ = 10^−4^, asymmetric migration rates across numerous selection coefficients (**C** and **G**, with $r_{AM}=r_{MF}$ = 10^−4^), and recombination rates (**D** and **H**) with a symmetric selection coefficient of 0.8 and *g*_non-reinf→reinf_ = *g*_reinf→non-reinf_ = 0.03. Complete or nontransient reinforcement is visible on the far right of figures **F** and **H**, indicated by the darkest green color, and the ∞ symbol in **F**. The amount of reinforcement is quantified as (*p*_[*z*, gen = x]_−*p*_[*z*,gen = 0]_) / *p*_[*z*,gen = 0]_, where *p_z_* equals the probability of being fertilized by nonmigrant sperm/pollen, scaled by the frequency of nonmigrant sperm/pollen. Time in generations is represented by “gen”. The data underlying this figure can be found in https://datadryad.org/stash/dataset/doi:10.5061/dryad.rjdfn2zf8.

We now show how varying parameter values influence the maximum amount (Fig 4A–4D) and duration (Fig 4E–4H) of reinforcement in the face of this conflict.

#### Reinforcement often requires strong selection

As selection on the locally adaptive allele intensifies, both the maximum extent (Fig 4A) and total duration (Fig 4E) of reinforcement increase. With symmetric selection and symmetric migration, selection on the local adaptation locus must be exceptionally strong for any reinforcement to evolve—e.g., even with *s* = 0.3, only very subtle reinforcement evolves for a very short time. However, other parameter choices—such as asymmetric migration or selection—can mediate the strength of selection required for reinforcement to evolve (see below).

With symmetric migration and asymmetric selection, the strength of selection in *non-reinf* (the population without the stylar incompatibility) generally has a greater effect on the extent and duration of reinforcement than does the strength of selection in *reinf* (Figs 4B and 4F and S3). This is because strong selection in *non-reinf* removes the migrant *MA* haplotype, minimizing opportunities for *M* to recombine onto the locally adapted *a* background.

#### The extent and symmetry of migration mediates reinforcement

With symmetric migration, intermediate migration rates always maximize the extent of reinforcement (Figs 4A and S3A), while the duration of reinforcement decreases with the migration rate (Figs 4E and S3B), regardless of the selection coefficient.

The effect of asymmetric migration on the extent of reinforcement highlights how migration mediates this sexual conflict. Migration from *non-reinf* to *reinf* favors reinforcement by increasing the number of maladapted immigrants available for heterospecific matings (Fig 4C). By contrast, increasing migration from *reinf* to *non-reinf* accelerates the introgression of the *M* allele into *non-reinf*, especially at higher migration rates, rapidly undermining reinforcement (Fig 4G). With unidirectional migration from *non-reinf* to *reinf*, substantial reinforcement can persist for prolonged time periods (Fig 4C and 4G).

#### Linkage between female barrier and male (in)compatibility alleles does not strongly impact the amount or duration of reinforcement

Contrary to classic results of reinforcement theory [10], linkage between the male and female (in)compatibility alleles, $r_{MF}$, has only a modest effect on the evolution of reinforcement. This result is seen across most selection coefficients and most values of $r_{AM}$ (Fig 4D and 4H; reproduced in S5A and S5D Fig). Reordering the loci in the model does not alter this outcome—i.e., the extent and duration of reinforcement is largely insensitive to $r_{MF}$ in models with loci in MFA order (S5B and S5E Fig).

Instead, linkage between the local adaptation locus, A, and either M or F loci are critical to the evolution of reinforcement. Marker order MAF highlights the impact of recombination between the components of the PMPZ incompatibility complex on both the duration and intensity of reinforcement (S5C and S5F Fig). While both $r_{AM}$ and $r_{FA}$ modulate the level of reinforcement (S5C Fig), the duration of reinforcement is independent of $r_{FA}$ (S5E Fig), and nearly completely determined by recombination between the male compatibility M and local adaptation locus $A,\;r_{AM}$. When A and M are tightly linked, substantial reinforcement can evolve and last for some time. The strength and duration of reinforcement drops, initially modestly, and then quite precipitously, as the recombination rate increases, with nearly no reinforcement evolving when A and M are unlinked (Fig 4D and 4H). Selection modulates this effect of recombination (S4 Fig); when selection is very strong (e.g., *s* > 0.6) some reinforcement can evolve, even when A and M are separated by up to a centiMorgan (i.e., *r*_*AM*_ = 0.01).

This result suggests that the rate of recombination between the local adaptation and male compatibility loci, $r_{AM}$, underlies the sexual conflict over reinforcement. When $r_{AM}$ is high, meaning A and M are loosely linked, *M* can more easily recombine onto the locally adapted *a* background, which facilitates its introgression into *non-reinf*. By escaping from the *A* background, *M* has greater long-term viability in *non-reinf* than it would if it remained associated with this locally maladaptive allele, increasing the male benefit to overcoming the incompatibility.

#### The presence of multiple unlinked local adaptation loci allows for (transient) reinforcement

Our results so far suggest that transient reinforcement by PMPZ incompatibilities requires tight linkage between loci underlying incompatibility and a single locus under divergent selection. However, the genetic architecture of local adaptation is often polygenic [27].

**Fig 5:**
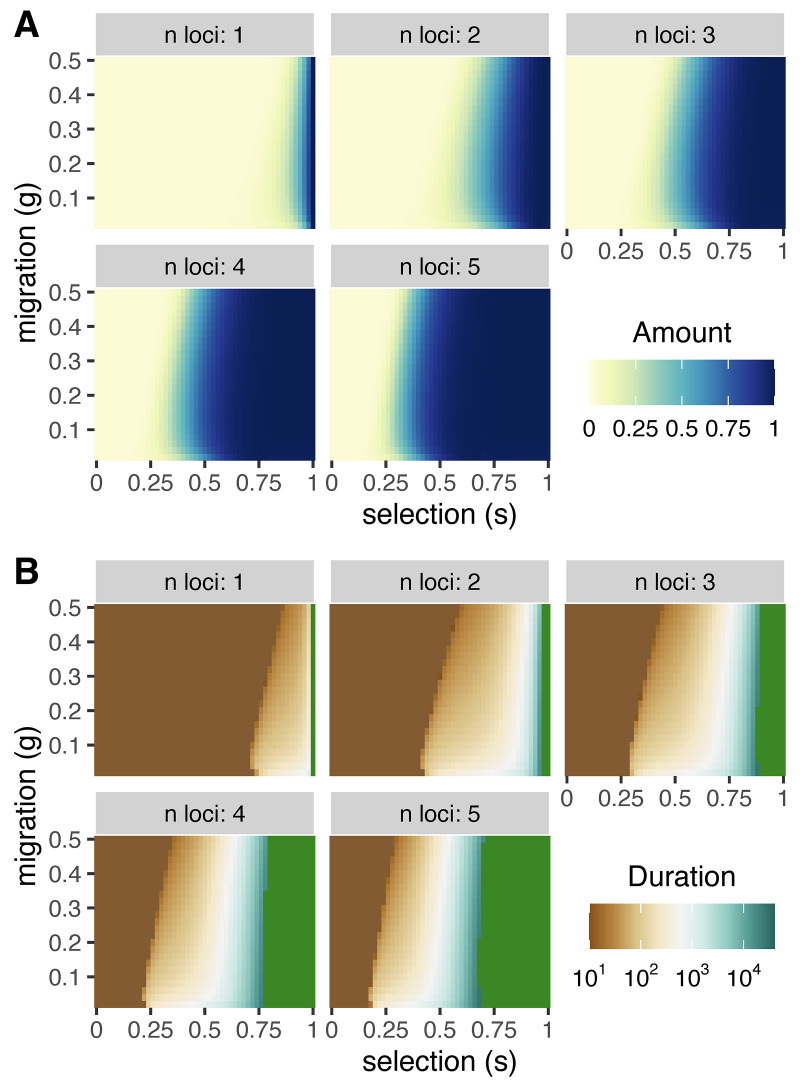
Oligogenic ecological selection. The maximum strength (**A**) and duration (**B**) of reinforcement with ecological selection at *n* loci, where all ecologically selected loci are unlinked to one another and to the gametophytic factor. The selection coefficient *s* against a maladaptive allele is multiplicative within and among loci—e.g., the fitness of an individual homozygous for the locally maladaptive allele at all *n* loci is (1−*s*)^2*n*^. Migration rate *g* is symmetric and recombination rate $r_{MF}=10^{-4}$. The data underlying this figure can be found in https://datadryad.org/stash/dataset/doi:10.5061/dryad.rjdfn2zf8.

We therefore investigate if weaker selection at more unlinked loci can allow reinforcement to transiently evolve by setting $r_{A_{1}M}$ to 0.5 and introducing up to 4 additional unlinked local adaptation A loci. Fig 5 shows that reinforcement can evolve when alternate alleles at numerous unlinked loci experience divergent selection in the 2 populations. This is consistent with recent work showing that, when numerous loci underlie reproductive isolation, selection on early-generation hybrids acts not against isolated loci, but on phenotypes underlain by pedigree relationships [28]. While the selection coefficients displayed are still quite large, this suggests that weaker selection at many loci could likely result in the transient evolution of reinforcement.

#### An opposing gametophytic factor does not stabilize reinforcement

To explore the possibility that the collapse of reinforcement could be prevented by the presence of distinct incompatibilities expressed in each population, we included a model in which both populations are “reinforcing” but by an independent set of loci. Although a second complementary incompatibility allows reinforcement to begin at lower selection intensities and slightly expands the parameter space for which reinforcement stably evolves (S6A Fig), reinforcement usually remains transient (S6B Fig).

#### A large cost of the male compatibility allele can stabilize reinforcement

We next ask how a cost to the male compatibility allele impacts the evolution of reinforcement; ignoring the question of how such a costly allele could have spread in *reinf*. For illustrative purposes, we limit our focus to exploration of parameters presented in Fig 2 and described in its legend.

Assigning a cost *s*_*M*_ to the *M* allele can lead to one of three qualitatively different outcomes (S7 Fig). If its cost is sufficiently small (*s*_*M*_<0.01 in our example), *M* rapidly and adaptively introgresses and undermines reinforcement as shown above. A larger cost to *M* (0.01<*s*_*M*_<0.08) results in an equilibrium level of reinforcement (i.e., partial reproductive isolation [29]) wherein the cost of *M* is balanced by a benefit of heterospecific fertilization to *M*-bearing pollen/sperm. An even larger cost to *M* (*s*_*M*_>0.08 in our example) prevents the introgression of this allele altogether, resulting in stable reinforcement. In sum, a sufficiently large cost to the male compatibility allele can stabilize the evolution of reinforcement, but a small cost does not.

## Discussion

For decades, researchers have presented theoretical and empirical challenges to the process of reinforcement, starting with a foundational paper by Felsenstein that identified recombination as a critical challenge to reinforcement [10]. Since then, a large body of theory has investigated the circumstances that permit or hinder the evolution of reinforcement (reviewed in [30]). Despite its potential role in hampering speciation [1], however, sexual conflict over hybridization has received relatively little attention in the literature. When it is mentioned, sexual conflict over hybridization is included only as a brief aside in papers concerning the role of sexual conflict in speciation more broadly [1,5,6].

Here, we identify the transient dynamics generated by sexual conflict over reinforcement and the evolutionary traces it leaves behind—namely the adaptive spread of female barriers in one species/population and the adaptive introgression of male compatibility alleles into the other. These results provide a rich set of predictions and interpretations of empirical patterns that were absent from previous game theoretic [1,5] and verbal [31] models.

In our model, sexual selection favors sperm/pollen traits that overcome a heterospecific female barrier. This poses a conflict: Females are selected to avoid the production of maladapted hybrids, while sperm (or pollen) that increase their fertilization success will generally be favored. The breakdown of reproductive isolation is marked by the rapid adaptive introgression of the male compatibility trait into *non-reinf*, following recombination onto the locally adapted haplotype. Back-migration of this allele into *reinf* hastens its fixation across populations. The final step in the model is the slow homogenization of the female barrier allele across demes, as the male compatibility allele fixes in both, rendering the female choice allele ineffective. Such homogenization of female preference does not occur when reinforcement is stable, and the male trait does not spread across both populations. Ultimately, we show that barriers acting at different stages of hybridization can affect how reinforcement proceeds. Below, we discuss the relationship of our results to previous theory, implications for the process of reinforcement, and empirical implications for hybridizing taxa, including *Z*. *mays* subspecies.

### Theoretical context and predictions for reinforcement

Previous models of reinforcement treated the sexes interchangeably [10] or assumed assortative mating under female control [32–34] (but see [8,9,35] for models with male choice) either by “matching” or “preference/trait” mechanisms of assortative mating [24]. Both matching and preference/trait models induce a trade-off between heterospecific and conspecific mating: a male with a trait favored by heterospecific females will have limited mating success with conspecific females.

While numerous studies have addressed the role of introgression in reinforcement (e.g., [36–40]), a distinguishing feature of our model is the mechanism of nonrandom fertilization, which we model as a PMPZ incompatibility functioning as a gametophytic factor in *Z*. *mays* [41] and PMPZ barriers in broadcast spawners (e.g., [42]). In this special class of preference/trait model, introgression of a male compatibility allele is facilitated by the fact that it does not prevent heterospecific mating—i.e., by definition, our model lacks a trade-off between con- and heterospecific mating (i.e., no reallocation *sensu*; [9]). As such, (in)compatibility-type mating interactions can result in the transient evolution of reinforcement, while other matching or preference/trait models cannot.

### Implications for reinforcement by pre- versus postmating barriers

Our model assumes that being attractive to one species does not come at a cost of being attractive to ones’ own species. How does this assumption likely map onto cases of reinforcement by pre- and postmating isolation mechanisms?

#### Postmating prezygotic isolation

To the extent that PMPZ barriers function similarly to those in our study, our model suggests transient reinforcement by PMPZ barriers. However, physical and/or biochemical properties of PMPZ interactions may minimize the opportunity for interspecific sexual conflict by enforcing a trade-off between overcoming a heterospecific barrier and successfully fertilizing conspecifics. This would allow for the evolutionary stability of reinforcement, consistent with our results from our model that showed that a sufficiently large cost to pollen/sperm compatibility could stabilize reinforcement. For example, as Howard [43] argued, and Lorch and Servedio [23] showed, a preference for conspecific sperm can stably evolve to minimize heterospecific fertilization. Thus, conspecific sperm precedence in competitive fertilization likely allows stable reinforcement by PMPZ isolation (as shown by, e.g., [44]). Likewise, mechanistic features of noncompetitive fertilization can also induce a trade-off between inter- and intraspecific crossing success. For example, if pollen must grow an optimal distance to fertilize an ovule (as observed in interspecific *Nicotiana* crosses) [45], success on both hetero- and conspecific styles is impossible.

#### Premating isolation

In principle, our model could apply to the reinforcement of premating isolation, as well as postmating isolation, so long as there is no trade-off in attractiveness to each species. There are numerous examples of premating traits that may increase heterospecific reproductive success without trading off conspecific attractiveness. For example, divergence in male competitive ability between two lineages of the common wall lizard (*Podarcis muralis*) results in asymmetric hybridization [46,47]. Likewise, loci underlying plumage traits appear to be asymmetrical and adaptively introgress from one to another subspecies of the red-backed fairywren, *Malurus melanocephalus* [48], presumably because this trait confers an advantage in extra-pair copulation due to sensory bias [49]. In a classic example of this phenomenon, plumage of the golden-collared manakin *Manacus vitellinus* appears to adaptively introgress into *Manacus candei* upon secondary contact [50], and this spread is likely mediated by female choice [51]. However, it does not appear that the female preference in any of these cases initially arose as a mechanism of reinforcement.

### Sexual conflict and sexual selection undermine reinforcement

Our model shows that the common phenomenon of sexual conflict (see [3] for examples), wherein male and female interests are misaligned, can erode reinforcement by PMPZ incompatibilities. This role for sexual conflict in removing species boundaries runs counter to the conventional role it is thought to play in speciation [1]. Previous theory [52] and experiments [53], as well as natural patterns of reproductive isolation [54,55] and diversification rates [56] suggest that independent coevolutionary arms races between male and female traits in two incipient species can pleiotropically result in behavioral or mechanical isolation. In this manner, intraspecific sexual conflict was thought to be an “engine of speciation” [57]. By contrast, we show that interspecific conflict between the sexes over fertilization hampers speciation. This highlights an underappreciated challenge to reinforcement by PMPZ barriers. Broadly, our results align with studies suggesting that incompatibilities, especially those with a transmission advantage [58], can adaptively introgress across species boundaries [59].

Servedio and Bürger [12] found that Fisherian sexual selection can undermine reinforcement. Specifically, they found that migration of female preference alleles provides a mating advantage to otherwise locally maladaptive heterospecific male traits by sexual selection, undermining reinforcement. In contrast, our model shows that the benefit of siring low fitness hybrids, when the alternative is missed fertilization opportunities that result in no offspring, can also undermine the evolution of reinforcement. Counter to Servedio and Bürger, we find that the female incompatibility remains at low frequency in the non-reinforcing species for a long time. As such, our models make contrasting predictions: Servedio and Bürger’s model predicts that alternative female preferences will coexist in both populations early in the evolutionary process, while our model predicts that female preference for the “wrong” species signal will only become common in both populations very late in the process of genetic exchange.

Our model can be seen as a specific instance of the lek paradox [60], as female preference ultimately erodes variation for a male trait. Following previously proposed resolutions of the lek paradox (e.g., [61]), Proulx [62] suggested that a female preference for an indicator of paternal fitness (e.g., sperm competitiveness) could act as a “one allele” mechanism of reinforcement (sensu; [10]). Under our model of sexual conflict, however, the lek paradox is ultimately unresolved. Rather, our model results in a male trait fixed in both species, which does not ultimately aid in assortative mating but is a mark of the “ghost of reinforcement past”.

### Empirical implications, predictions, and interpretation of current observations

Our model, based on a well-characterized PMPZ incompatibility, shows that reinforcement by such mechanisms is precarious. As such, to the extent that such barriers do not incur a trade-off between conspecific and heterospecific fertilization success, we predict that reinforcement by PMPZ barriers should be rare. Thus, the finding that gametic isolation in broadcast spawners is not the product of reinforcement [63], as well as meta-analyses showing that PMPZ isolation does not differ between sympatric and allopatric species pairs in *Drosophila* [64] or across 3 angiosperm genera [65], are consistent with our model. Still, negative evidence is not necessarily evidence for the negative.

As such, the few documented cases in which PMPZ barriers are reinforced allow for better evaluation of our predictions. Specifically, we predict that reinforcement by PMPZ barriers should often involve certain characteristics, which are consistent with the empirical literature. These include the following: (1) recent sympatry, so that the male barrier has not yet increased in frequency (e.g., [66]); (2) a trade-off between male success in overcoming inter- and intraspecific postmating barriers, as is found in preference/trait mechanisms (e.g., [44]); (3) unidirectional gene flow (e.g., [67]); and/or (4) exceptionally strong postzygotic isolation, such that gene flow is very rare, as seen in *Drosophila yakuba* and *Drosophila santomea* (e.g., [64]). However, reinforcement by PMPZ isolation in *D*. *yakuba* and *D*. *santomea* is difficult to reconcile with our model, as the pair have a stable hybrid zone, no evidence of conspecific sperm precedence, and bidirectional hybridization [68]. Nonetheless, our model suggests a plausible evolutionary mechanism for existing cases of reinforcement by PMPZ isolation and generates specific hypotheses to be tested.

### Predictions for maize and teosinte

*Zea mays* subsp. *mays* and *Zea mays* subsp. *mexicana* grow in close sympatry and hybridize [69,70]. Evidence for genome-wide selection against admixture, despite adaptive introgression of some teosinte loci into maize [71], is consistent with the idea that hybrids are often disfavored—perhaps because hybrids are removed from maize fields by anthropogenic weeding, and maize traits expressed in teosinte environments are likely maladaptive [72,73] (although clear cases of adaptive introgression and deliberate hybridization by farmers exist).

This system has all the ingredients necessary for reinforcement—the occurrence of gene flow, the presence of a stylar incompatibility in teosinte sympatric with maize, and the reduced but nonzero fitness of hybrids. However, the elevated pollen discrimination exhibited by highland teosinte sympatric with maize [15–18] is surprisingly ineffective in preventing fertilization by sympatric maize landraces [21,22], against whom selection for reinforcement should be strongest.

Our model explains this observation as the initial evolution of reinforcement (i.e., a stylar barrier sweeps through teosinte) followed by the adaptive introgression of teosinte pollen compatibility alleles into maize. Notably, alternative explanations for this pattern are insufficient. For example, this pattern is not simply attributable to the loss of isolation upon secondary contact, because allopatric teosinte do not reject maize pollen [21,22]. Nor can this be explained by complex speciation, in which teosinte sympatric with maize would be more recently diverged from maize than are allopatric teosinte, as this is incompatible with both genetic evidence and the history of maize domestication [74]. We suggest that in most sympatric populations, at most gametophytic factors, the stylar fertilization barrier (the *F* allele) rapidly swept through teosinte (Phase 1 in Fig 2), and the pollen compatibility allele (*M*) adaptively introgressed into sympatric highland maize landraces (Phase 2 in Fig 2).

### Caveats

Our model made many simplifications and abstractions for tractability and generality. Most notably, we assumed only two populations, a single gametophytic factor, and a simple multiplicative fitness function across a small number of divergently selected loci. Our results show that the architecture of adaptive differentiation and the linkage between locally adaptive alleles and PMPZ incompatibilities modulate the rise and fall of reinforcement. Across taxa, adaptive differentiation can be controlled by few [75–77] or many [78] loci, and linkage between locally adaptive alleles and PMPZ incompatibilities is biologically variable and rarely known. In maize, evidence is mixed—one gametophytic factor *tcb1* is tightly linked to a domestication locus *su1* [79,80], which likely experiences divergent selection. However, two other known gametophytic factors are far from loci under strong divergent selection.

We further assume that the male compatibility allele is initially common and stylar incompatibility is initially rare in *reinf*, and we do not address the origins of gametophytic factors. While the initial divergence of these alleles is outside the scope of our model, it could be explained by pleiotropy, selection to prevent polyspermy (a known risk to embryo viability in maize) [81], or Fisherian runaway selection (as proposed for gametophytic factors by Jain) [82]. Pleiotropy may explain the initial evolution of gametophytic factors in *Z*. *mays*, as they are members of the multifunction pectin methylesterase (PME) and PME inhibitor (PMEI) gene families [15–17,83] and could be favored by mechanisms related to other functions. Notably, a subclass of PMEs contain both PME and PMEI domains, providing a potential explanation for tight linkage of the *M* and *F* alleles [84].

## Conclusions

We find that considering the role of sexual conflict—a mismatch between optimal fertilization outcomes of each sex—in reinforcement generates novel predictions and may explain numerous patterns in nature. Our results are particularly relevant to potential cases of reinforcement by gamete recognition in plants, as well as broadcast spawners (e.g., Lysin/VERL in abalone [85] or Bindin/EBR1 in sea urchins [86]), and even to cases of internal fertilization in which premating isolation is inefficient, costly, or otherwise unpreferred [87]. In these situations, we predict that reinforcement by PMPZ will be rare, transient, or involve a trade-off between heterospecific and conspecific fertilization (i.e., some mechanism of reallocation). Finally, although our model is developed specifically for interactions between haploid male gametes and diploid females, similar dynamics could arise for premating barriers with a similar genetic architecture and lacking reallocation of male reproductive effort.

## Materials and methods

### Quantifying reinforcement and its duration

We summarized our results by quantifying the duration and maximum extent of reinforcement. We quantified the amount of reinforcement at generation *g* as $(p_{\left[z,\mathrm{gen}=g\right]}-p_{\left[z,\mathrm{gen}=0\right]})/p_{\left[z,\mathrm{gen}=0\right]}$. Where *p*_*z*_ equals the probability of being fertilized by nonmigrant sperm/pollen, scaled by the frequency of nonmigrant sperm/pollen. We quantified the duration of reinforcement as the number of generations for which the amount of reinforcement was greater than 0.05.

### Partitioning selection

All selection for or against the female incompatibility allele, *F*, is indirect, as it does not itself impact fitness; i.e., selection impacts the frequency of an allele at the F locus, not because of its effect on fitness, but because of its genetic background (i.e., linkage disequilibrium between *F* and *A*). Each generation, some of the LD between *F* and *A* is immediately attributable to either (a) population structure, historical events, etc. (primarily by *cis*-LD), which we call “incidental selection” or (b) to the causal effect of the *F* allele in generating genetic associations by the gametes permitting fertilization (primarily via *trans*-LD; see subsection The generation of trans linkage disequilibrium during fertilization in the Mathematical Appendix S3), which we call “selection for reinforcement”. See [24] for a discussion of how LD in *cis* and *trans* contribute to Fisherian sexual selection. We developed this new terminology because “linked selection” and “indirect selection” are insufficient in distinguishing these causal forces.

Thus, the change in frequency in *F* is attributable to both its circumstance (“incidental selection”) and its causal effect on generating a nonrandom association (“selection for reinforcement"). We aim to partition total selection for (or against) the *F* allele into incidental selection (unrelated to the effect of *F* on nonrandom fertilization, *Δp*_*F*,Incidental_) and selection for reinforcement (the causal effect of *F* on its selective trajectory, *Δp*_*F*,Reinforcement_). In this exercise, we ignore the change in frequency of paternally derived genotypes (which includes migration, fertilization, and selection), as none of this change is plausibly attributable to the *F* allele. We include the subscript *mat* with each variable to remind readers we are focused on the maternally derived *F* alleles.

We first compute the difference in allele frequency between maternally derived haplotypes in offspring after versus before selection as *Δp*_*F*,mat_ directly from our results: *Δp*_*F*,mat_ = *p*_*F*,mat-derived after sel_−*p*_*F*,mom_. We then decompose *Δp*_*F*,mat_ into components of reinforcing and incidental selection:

$$\Delta p_{F,\mathrm{mat}}=\Delta p_{F,\mathrm{mat},\;\mathrm{Reinforcement}}+\Delta p_{F,\mathrm{mat},\;\mathrm{Incidental}}$$ (2)

Each generation, we find *Δp*_*F*,mat, Incidental_ by calculating *Δp*_*F*,mat_ under the counterfactual case of random fertilization. We then find the change in frequency of *F* by selection for reinforcement *Δp*_*F*,mat, Reinforcement_ by rearranging Eq 2.

#### Computer code

All code is written in R [88] and is available at https://github.com/carushworth/gameto-theory. We generated figures with the ggplot2 and cowplot packages [89,90] and used the dplyr package to process numeric results [91].

## Dryad DOI

10.5061/dryad.rjdfn2zf8 [92]

## Supporting information

S1 Text Pseudocode for our iteration. A detailed description of our model.
(DOCX)

S2 Text Traditional notation for gametophytic factors. Our model is inspired by gametophytic factors in that underlie PMPZ barriers between *Zea mays* subspecies. We connect our model to the traditional terminology that describes gametophytic factors.
(DOCX)

S3 Text Mathematical appendix. We derive key analytical results from our model.
(DOCX)

S1 FigThe initial frequency of *M* in the reinforcing species does not influence qualitative results.The impact of variability in the initial frequency of sperm/pollen compatibility allele, *M*, in *reinf*, on the transient reinforcement of postmating prezygotic isolation. All lines overlap. Parameter values: Selection — *s_reinf_* = *s_non-reinf_* = 0.75. Migration — (*g*_non-reinf→reinf_ = *g*_*reinf*→*non-reinf*_ = 0.1). Recombination — $r_{AM}=r_{MF}$ = 0.0001. Allele frequencies — *f_M0,reinf_* = displayed by color, *f_M0,non-reinf_* = 0, *f_F0,reinf_* = 0.01, *f_F0,non-reinf_* = 0. The data underlying this figure can be found in https://datadryad.org/stash/dataset/doi:10.5061/dryad.rjdfn2zf8.(EPS)

S2 FigFemale choosiness alters strength of reinforcement.We allow for an imperfect barrier (i.e., variation in female choice) by allowing females with fertilization barrier genotypes to be fertilized by a given haplotype, *k*, with probability $x_{k}=\frac{p_{k}(1-\delta_{k}c)}{\sum x_{k}}$, where *p*_*k*_ is the frequency of haplotype *k* in pollen after fertilization. *δ*_*k*_ equals 0 for compatible sperm/pollen grains and 1 for incompatible sperm/pollen grains. *c*, the efficacy of the barrier, is colored in the plot above. Parameter values: Selection—*s_reinf_* = *s_non-reinf_* = 0.75. Migration—(*g*_non-reinf→reinf_ = *g*_*reinf*→*non-reinf*_ = 0.1). Recombination—$r_{AM}=r_{MF}=0.0001$. Allele frequencies—*f*_*M*0,reinf_ = 1, *f*_*M*0,non-reinf_ = 0, *f*_*F*0,reinf_ = 0.01, *f*_*F*0,non-reinf_ = 0. The data underlying this figure can be found in https://datadryad.org/stash/dataset/doi:10.5061/dryad.rjdfn2zf8.(EPS)

S3 FigThe impact of asymmetric selection on the extent (A) and duration (B) of reinforcement.Reinforcement strength and duration are estimated over a range of symmetric migration rates with $r_{AM}=r_{MF}=10^{-4}$. The data underlying this figure can be found in https://datadryad.org/stash/dataset/doi:10.5061/dryad.rjdfn2zf8.(EPS)

S4 Fig The impact of linkage on the extent (A) and duration (B) of reinforcement. Reinforcement strength and duration are estimated over a range of symmetric selection coefficients. *g*_non-reinf→reinf_ = *g*_reinf→non-reinf_ = 0.03. The data underlying this figure can be found in https://datadryad.org/stash/dataset/doi:10.5061/dryad.rjdfn2zf8.
(EPS)

S5 FigLocus order impacts the amount and duration of reinforcement.Top row (**A–C**) is reinforcement amount; bottom row (**D–F**) is duration, as estimated under different marker orders. Default marker order is AMF: amount (**A**); duration (**D**). Marker order MFA: amount (**B**); duration (**E**). Marker order MAF: amount (**C**); duration (**F**). Shown are results with a symmetric selection coefficient of 0.8 and migration *g*_non-reinf→reinf_ = *g*_reinf→non-reinf_ = 0.01. The data underlying this figure can be found in https://datadryad.org/stash/dataset/doi:10.5061/dryad.rjdfn2zf8.(EPS)

S6 Fig Asymmetrical variation in female preference does not underlie transience of reinforcement. Incorporating a second gametophytic factor in the *non-reinf* does not qualitatively change the amount (**A**) or duration of reinforcement (**B**), although reinforcement begins at lower intensities of selection and reaches completion across more selection coefficients. The data underlying this figure can be found in https://datadryad.org/stash/dataset/doi:10.5061/dryad.rjdfn2zf8.
(EPS)

S7 FigIntroducing a cost (*s*_*M*_) to the male compatibility allele impacts the evolution of conflict over reinforcement.(**A**) The frequency of the *M* allele in *non-reinf* over time as a function of the additive cost of the allele (*s*_*M*_ noted by color, with several values labeled for clarity). The frequency of the *M* allele in *non-reinf* (**B**) and the extent of reinforcement (**C**) after 10,000 generations (taken to be the equilibrium value, as evidenced by S7A Fig). Results are plotted on a *log*_10_ scale for clarity, though numbers are nontransformed. Dashed grey lines in (**B**) and (**C**) note the values (0.0125 and 0.08) at which we transition from transient reinforcement to a polymorphic equilibrium, and then to complete and stable reinforcement, respectively. All measures describe populations after selection and before recombination. This figure illustrates a single set of parameter values with 1 adaptive locus. Selection: *s*_reinf_ = *s*_non-reinf_ = 0.75; Migration: *g*_non-reinf→reinf_ = *g*_reinf→non-reinf_ = 0.1; Recombination: $r_{AM}=r_{MF}=0.0001$; Allele frequencies: *f*_*M*0,reinf_ = 1, *f*_*M*0,non-reinf_ = 0, *f*_*F*0,reinf_ = 0.01, *f*_*F*0,non-reinf_ = 0. The data underlying this figure can be found in https://datadryad.org/stash/dataset/doi:10.5061/dryad.rjdfn2zf8.(EPS)

## Abbreviations

LD: linkage disequilibrium
PME: pectin methylesterase
PMEI: PME inhibitor
PMPZ: postmating prezygotic
