# Supplementary material for: Conflict over fertilization underlies the transient evolution of reinforcement
Source: PLoS Biol. 2022 Oct 13;20(10):e3001814. doi: 10.1371/journal.pbio.3001814 (PMC9560609; doi:10.1371/journal.pbio.3001814)
Supplement: S1 Text — A detailed description of our model. (DOCX) [file pbio.3001814.s001.docx]

## Supp. Text S1: Pseudo-code for our model

We forward iterated genotype frequencies over time, not by iterating a numerical equation, but rather by deterministic programmatic iteration of a the evolutionary process. R scripts are included as a supplemental file, but here we provide pseudo-code to describe the process to the reader.

1. **Initiation:** Beginning with user-specified initial allele frequencies in each population, as well as additional parameters (e.g. the number of unlinked, local adaptation loci), we build initial genotype frequencies, assuming linkage equilibrium and random mating within each population.
2. **Iteration:** Every generation, we iterate the evolutionary process, for both populations — one population at a time — by
   1. *Meiosis in males:* Generating haploid sperm/pollen grains from diploid genotypes by meiosis, using genotype frequencies in the previous generation.
   2. *Migration:* Making a sperm/pollen pool with haplotype frequencies $L_{ix}^{'}=L_{ix}-m\left( L_{ix}-L_{iy} \right)$. Recall that $m$ denotes the proportion of sperm/pollen in population $x$ which migrated from population $y$.
   3. *Pollination and Fertilization:* We assume that, after migration, sperm/pollen from the falls randomly on styles (or female gametes) within the population, so the frequency of mating between a given sperm/pollen haplotype and female genotype (with frequencies from this population after the conclusion of the previous generation) is their product. Fertilization is non-random, with $c$ indicating the strength of discrimination against incompatible $m$ alleles. For a given diploid female genotype, the frequency of paternal genomes with alleles $A$ and $a$ are $p_{A}$ and $p_{a}\times\left( 1-c \right)$, respectively, with each divided by the proportion of sperm/pollen compatible with that female genotype (all pollen for $ff$ genotypes and $\left( 1-c \right)\times p_{M\text{pollen after migration}}$ for $Ff$ and $FF$ genotypes).
   4. *Female meiosis and syngamy:* Finally, females undergo meiosis and zygotes are generated at random, conditional on compatibility between female/stylar genotypes and sperm/pollen haplotype (above).
   5. *Selection:* We count the number of alleles at all $\mathcal{A}$ loci that mismatch the environment, $n_{\text{maladapt}}$, and calculate the fitness of each genotype as $\left( 1-n_{\text{maladapt}} \right)^{s}$. We calculate mean fitness as the product of genotype fitness and genotype frequency, summed over all genotypes. Finally, we find the frequency of each genotype after selection as the product of its frequency before selection and its fitness, divided by mean fitness.
   6. *Return summary:* We return a vector of genotype frequencies, as well as the extent of reinforcement in this population in this generation (as described in the Methods). Optional returns are available (e.g. partitioning allele frequency change across the life cycle, and evaluating "counterfactual scenarios" for selection for reinforcement and linked selection on $F$), which we only used for a few exemplary scenarios to minimize computational and storage expense.
   7. *Repeat or conclude:* After conducting the steps above for both populations, we store the new genotypes’ frequencies, as well as other quantities of interest. The simulation then returns to (a) if stopping criteria have not been met, or concludes if so. By default, stopping criteria are: simulation length of at least 1000 generations and genotype frequencies at equilibrium (identified when the sum of the absolute value of genotype frequency change across all phased genotypes in both populations is less than 0.00001).
3. **Conclusion:** At the conclusion of the simulation, we return phased $\mathcal{AMF}$ genotype frequencies after selection in each population for every generation, the extent of reinforcement every generation, as well as optional summaries mentioned above (see code for all options).
