# Supplementary material for: Conflict over fertilization underlies the transient evolution of reinforcement
Source: PLoS Biol. 2022 Oct 13;20(10):e3001814. doi: 10.1371/journal.pbio.3001814 (PMC9560609; doi:10.1371/journal.pbio.3001814)
Supplement: S2 Text — Our model is inspired by gametophytic factors in that underlie PMPZ barriers between Zea mays subspecies. We connect our model to the traditional terminology that describes gametophytic factors. (DOCX) [file pbio.3001814.s002.docx]

## Supp. Text S2: Notation for gametophytic factors

Our notation throughout differs from the existing literature on gametophytic factors. In this literature, each of the three known gametophytic factors (Ga1, Ga2, and Tcb1) is represented as a single locus with three haplotypes (e.g. [1]). The “strong”’ haplotype, indicated by *s* or *S*, contains both functional alleles from this study ($F$ and $M$). The haplotype with only the pollen-expressed compatibility allele is referred to as *m*, and is equivalent to the *Mf* genotype in the present study. The non-functional allele is referred to in the literature in lowercase (e.g. ga1, ga2, tcb1), and is equivalent to the *mf* genotype in our study. The *mF* genotype is not known in the wild.

# References

1. Lauter AN, Muszynski MG, Huffman Rd, Scott MP. 2017. A Pectin Methylesterase ZmPme3 Is Expressed in Gametophyte factor1-s (Ga1-s) Silks and Maps to that Locus in Maize (*Zea mays* L.). *Frontiers in Plant Science* 8: 1926.
