# Supplementary material for: Conflict over fertilization underlies the transient evolution of reinforcement
Source: PLoS Biol. 2022 Oct 13;20(10):e3001814. doi: 10.1371/journal.pbio.3001814 (PMC9560609; doi:10.1371/journal.pbio.3001814)
Supplement: S3 Text — We derive key analytical results from our model. (DOCX) [file pbio.3001814.s003.docx]

## Supp. Text S3: Mathematical Appendix

We present standard recursion equations to provide some intuition for our results, but note that we did not use these recursions to generate our figures, which come from a iteration of the process rather than an explicit recursion of 27 unphased (64 phased) genotype frequencies in two non-randomly mating populations. Nor do we provide quasi-linkage equilibrium or Hardy-Weinberg equilibrium approximations as our focus is on the exact results provided in the main text. As such, the equations below, which largely recapitulate standard results from population genetic theory, are meant to provide some mathematical intuition for our results.

### Migration

All alleles change in frequency by migration in the same manner. Allele frequencies in pollen at the $i^{th}$ locus in the focal population $x$ after migration, $L_{ix}^{'}$, equal

$L_{ix}^{'}=L_{ix}-g_{\text{y}\to\text{x}}\left( L_{ix}-L_{iy} \right)$ (S1)

Where the prime ${}^{'}$ denotes the frequency in sperm/pollen after sperm/pollen migration and $L_{iy}$ is the allele frequency in the other population. From Eq S1, the change in allele frequency at the $i^{th}$ locus in sperm/pollen grains by sperm/pollen migration equals $\Delta_{L_{ix}^{'}\text{ migration}}=-g_{\text{y}\to\text{x}}\left( L_{ix}-L_{iy} \right)$, a standard result in population genetics.

### Fertilization

We assume both no sperm/pollen limitation, and an infinite supply of sperm/pollen, such that the frequency of a sperm/pollen haplotype on a female/style equals the frequency of this sperm/pollen haplotype in the local environment. Under this model, selection during fertilization changes the frequency of alleles in paternally-derived haplotypes relative to their frequency in sperm/pollen after migration, and does not impact allele frequencies of maternally-derived haplotypes.

After mating, the frequency of paternally-derived haplotypes bearing the sperm/pollen allele is $p_{M^{''}}$, with the double prime denoting that frequencies are calculated after migration and fertilization. This value depends on the relative advantage of the sperm/pollen compatibility allele on a given female/stylar genotype, $\tilde{c_{i}}$ (that is, the relative fitness of the compatible sperm/pollen haplotype $M$ on a the $i^{th}$ female genotype), weighted by the frequency of the $i^{th}$ diploid female genotype at the $\mathcal{F}$ locus (i.e. before migration), $p_{i}$:

$p_{M^{''}}=\sum\tilde{c_{i}}p_{i}$ (S2)

Where $\tilde{c_{i}}=1/\left( 1-c_{i}p_{m^{'}} \right)$. If the female (stylar) barrier is dominant, $c_{FF}=c_{Ff}=c$ and $c_{ff}=0$, and $p_{M^{''}}=\left( 1-p_{ff} \right)\frac{p_{M^{'}}}{1-cp_{m^{'}}}+p_{ff}p_{M^{'}}$. We can then find $\Delta p_{\text{ fertilization}}$ as

$\begin{matrix} \Delta p_{\text{ fertilization}} & =p_{M^{''}}-p_{M^{'}} \\ & =\left( 1-p_{ff} \right)\left( \frac{p_{M^{'}}}{1-cp_{m^{'}}}-p_{M^{'}} \right)+p_{ff}\left( p_{M^{'}}-p_{M^{'}} \right) \\ & =\left( 1-p_{ff} \right)\left( \frac{p_{M^{'}}}{1-cp_{m^{'}}}-p_{M^{'}} \right) \\ & =\left( 1-p_{ff} \right)\left( \frac{p_{M^{'}}}{1-cp_{m^{'}}}-\frac{p_{M^{'}}\left( 1-cp_{m^{'}} \right)}{1-cp_{m^{'}}} \right) \\ & =\left( 1-p_{ff} \right)\left( \frac{cp_{M^{'}}p_{m^{'}}}{1-cp_{m^{'}}} \right) \end{matrix}$ (S3)

Equation S3 is the standard allele frequency change from population genetics weighted by the frequency with which selection can operate — i.e. the frequency of incompatible females.

#### Linked selection during fertilization.

We now consider the change in frequency of an allele ($A$ or $F$, described below as $X$ which includes both alleles) in linkage disequilibrium with the sperm/pollen incompatibility allele $M$ due to the fertilization advantage of $M$.

After mating, we calculate the frequency of paternally-derived haplotypes bearing this linked allele, $p_{X^{''}}$, where the double prime indicates that the frequency is calculated after both migration and mating. This quantity depends on the advantage of the sperm/pollen compatibility allele on a given female (stylar) genotype and the statistical association between alleles $M$ and $X$ (described by gametic phase linkage disequilibrium, $D_{MX}$, which is the covariance between $M$ and $X$ alleles in sperm/pollen after sperm/pollen migration), weighted by the marginal frequency of the diploid genotype at the $\mathcal{F}$ locus in females/styles (i.e. before migration), $p_{i}$.

$p_{X^{''}}=p_{FF}\times\frac{p_{X}^{'}+c\left( D_{MX}^{'}+p_{x}^{'}p_{m}^{'} \right)}{1-cp_{m}^{'}}+p_{Ff}\times\frac{p_{X}^{'}+c\left( D_{MX}^{'}+p_{x}^{'}p_{m}^{'} \right)}{1-cp_{m}}+p_{ff}\times\frac{p_{X}^{'}}{1}$ (S4)

The change in frequency of an allele linked to $\mathcal{M}$ between pollination (mating) and fertilization is therefore

$\Delta p_{X\text{ fertilization}}=\left( 1-p_{ff} \right)\frac{c\times D_{MX}^{'}}{1-cp_{m}^{'}}$ (S5)

As such, the alleles in positive linkage disequilibrium with the sperm/pollen compatibility allele $M$ always increase in frequency during fertility selection because of the fertilization advantage, so long as there are some incompatible females/styles in the population. Because linkage disequilibrium between $M$ and $F$, as well as $M$ and $A$, is never less than zero, all teosinte alleles increase in frequency in both populations by fertility selection (Fig 3B).

#### The generation of trans linkage disequilibrium during fertilization.

We begin with two diverged populations, fixed for alternative alleles at local adaptation and sperm/pollen compatibility loci and with different allele frequencies at the female/stylar incompatibility. As such, LD between sperm/pollen compatibility and locally adaptive alleles is initially very large, and is broken down by migration and recombination.

This incompatibility system generates *trans* LD between sperm/pollen and female (stylar) loci — that is, compatibility rules generate a statistical association between paternally-derived $M$ alleles and maternally derived $F$ alleles. Before selection, this *trans* LD equals

$D_{MF,trans}=\frac{cp_{m}^{'}p_{M}^{'}p_{F}p_{ff}}{1-cp_{m}^{'}}$ (S6)

Clearly, this also indirectly generates *trans* LD between maternally-derived female incompatibilities and loci in LD with the sperm/pollen compatibility allele (or deviations from Hardy-Weinberg equilibrium at the $F$ locus). *Trans* LD is converted to *cis* LD by recombination.

### Local adaptation

Genotype frequencies at the local adaptation locus $\mathcal{A}$ after selection follow the standard population genetic equation $p_{i}^{'}=p_{i}w_{i}/\overline{w}$.

With multiplicative fitness effects, at a single local adaptation locus in the population in which $A$ is favored, genotypic fitnesses are: $w_{AA}=1$, $w_{Aa}=\left( 1-s \right)$, and $w_{aa}=\left( 1-s \right)^{2}$, and mean fitness equals $\overline{w}=1-2p_{a}s+s^{2}p_{aa}$. After selection, genotype frequencies are:

$\begin{matrix} p_{AA,\text{next gen}} & =\frac{p_{AA}^{‴}}{\overline{w}} \\ p_{Aa,\text{next gen}} & =\frac{p_{Aa}^{‴}\left( 1-s \right)}{\overline{w}} \\ p_{aa,\text{next gen}} & =\frac{p_{aAa}^{‴}\left( 1-s \right)^{2}}{\overline{w}} \end{matrix}$ (S7)

and the change in genotype frequencies between fertilization and viability selection are

$\begin{matrix} \Delta p_{AA,\text{next gen}} & =\frac{p_{AA}^{‴}-\overline{w}p_{AA}^{‴}}{\overline{w}}=\frac{sp_{AA}^{‴}\left( 2p_{a}^{‴}-sp_{aa}^{‴} \right)}{\overline{w}} \\ \Delta p_{Aa,\text{next gen}} & =\frac{p_{Aa}^{‴}\left( 1-s \right)-\overline{w}p_{Aa}}{\overline{w}}=-\frac{sp_{Aa}^{‴}\left( 1-2p_{a}^{‴}+sp_{aa}^{‴} \right)}{\overline{w}} \\ \Delta p_{aa,\text{next gen}} & =\frac{p_{aAa}^{‴}\left( 1-s \right)^{2}-\overline{w}p_{aa}^{‴}}{\overline{w}}=\frac{-2sp_{aa}^{‴}\left( 1-p_{a}^{‴}-s\left( 1-p_{aa}^{‴} \right)/2 \right)}{\overline{w}} \end{matrix}$ (S8)

.

With multiplicative fitness, the locally maladapted allele always decreases by direct selection, regardless of allele frequency or inbreeding coefficient.

#### Linked selection during local selection.

After fertilization, selection directly changes the frequency of the local adaptation loci as described above. This also changes the frequency of loci statistically associated with the locally adapted allele. The change in frequency of an allele, $X$ (e.g. $F$ or $M$), linked to $\mathcal{A}$ by linked selection is determined by the selection coefficient $s$ and zygotic LD, and equals:

$\begin{matrix} \Delta X= & \frac{s\left( 2D_{XX,A}+D_{Xx,A} \right)+s^{2}\left( D_{XX,aa}+D_{Xx,aa}/2 \right)}{1-2p_{a}s+s^{2}p_{aa}} \\ & \text{where} \\ D_{XX,A} & =p_{XX}p_{a}-p_{XXa} \\ D_{Xx,A} & =p_{Xx}p_{a}-p_{Xxa} \\ D_{XX,aa} & =p_{XXaa}-p_{XX}p_{aa} \\ D_{Xx,aa} & =p_{Xxaa}-p_{Xx}p_{aa} \end{matrix}$ (S9)

## Supp. Text S3: Mathematical Appendix

We present standard recursion equations to provide some intuition for our results, but note that we did not use these recursions to generate our figures, which come from a iteration of the process rather than an explicit recursion of 27 unphased (64 phased) genotype frequencies in two non-randomly mating populations. Nor do we provide quasi-linkage equilibrium or Hardy-Weinberg equilibrium approximations as our focus is on the exact results provided in the main text. As such, the equations below, which largely recapitulate standard results from population genetic theory, are meant to provide some mathematical intuition for our results.

### Migration

All alleles change in frequency by migration in the same manner. Allele frequencies in pollen at the $i^{th}$ locus in the focal population $x$ after migration, $L_{ix}^{'}$, equal

$L_{ix}^{'}=L_{ix}-g_{\text{y}\to\text{x}}\left( L_{ix}-L_{iy} \right)$ (S1)

Where the prime ${}^{'}$ denotes the frequency in sperm/pollen after sperm/pollen migration and $L_{iy}$ is the allele frequency in the other population. From Eq [[eqn:mig]](#eqn:mig), the change in allele frequency at the $i^{th}$ locus in sperm/pollen grains by sperm/pollen migration equals $\Delta_{L_{ix}^{'}\text{ migration}}=-g_{\text{y}\to\text{x}}\left( L_{ix}-L_{iy} \right)$, a standard result in population genetics.

### Fertilization

We assume both no sperm/pollen limitation, and an infinite supply of sperm/pollen, such that the frequency of a sperm/pollen haplotype on a female/style equals the frequency of this sperm/pollen haplotype in the local environment. Under this model, selection during fertilization changes the frequency of alleles in paternally-derived haplotypes relative to their frequency in sperm/pollen after migration, and does not impact allele frequencies of maternally-derived haplotypes.

After mating, the frequency of paternally-derived haplotypes bearing the sperm/pollen allele is $p_{M^{''}}$, with the double prime denoting that frequencies are calculated after migration and fertilization. This value depends on the relative advantage of the sperm/pollen compatibility allele on a given female/stylar genotype, $\tilde{c_{i}}$ (that is, the relative fitness of the compatible sperm/pollen haplotype $M$ on a the $i^{th}$ female genotype), weighted by the frequency of the $i^{th}$ diploid female genotype at the $\mathcal{F}$ locus (i.e. before migration), $p_{i}$:

$p_{M^{''}}=\sum\tilde{c_{i}}p_{i}$ (S2)

Where $\tilde{c_{i}}=1/\left( 1-c_{i}p_{m^{'}} \right)$. If the female (stylar) barrier is dominant, $c_{FF}=c_{Ff}=c$ and $c_{ff}=0$, and $p_{M^{''}}=\left( 1-p_{ff} \right)\frac{p_{M^{'}}}{1-cp_{m^{'}}}+p_{ff}p_{M^{'}}$. We can then find $\Delta p_{\text{ fertilization}}$ as

$\begin{matrix} \Delta p_{\text{ fertilization}} & =p_{M^{''}}-p_{M^{'}} \\ & =\left( 1-p_{ff} \right)\left( \frac{p_{M^{'}}}{1-cp_{m^{'}}}-p_{M^{'}} \right)+p_{ff}\left( p_{M^{'}}-p_{M^{'}} \right) \\ & =\left( 1-p_{ff} \right)\left( \frac{p_{M^{'}}}{1-cp_{m^{'}}}-p_{M^{'}} \right) \\ & =\left( 1-p_{ff} \right)\left( \frac{p_{M^{'}}}{1-cp_{m^{'}}}-\frac{p_{M^{'}}\left( 1-cp_{m^{'}} \right)}{1-cp_{m^{'}}} \right) \\ & =\left( 1-p_{ff} \right)\left( \frac{cp_{M^{'}}p_{m^{'}}}{1-cp_{m^{'}}} \right) \end{matrix}$ (S3)

Equation S3 is the standard allele frequency change from population genetics weighted by the frequency with which selection can operate — i.e. the frequency of incompatible females.

#### Linked selection during fertilization.

We now consider the change in frequency of an allele ($A$ or $F$, described below as $X$ which includes both alleles) in linkage disequilibrium with the sperm/pollen incompatibility allele $M$ due to the fertilization advantage of $M$.

After mating, we calculate the frequency of paternally-derived haplotypes bearing this linked allele, $p_{X^{''}}$, where the double prime indicates that the frequency is calculated after both migration and mating. This quantity depends on the advantage of the sperm/pollen compatibility allele on a given female (stylar) genotype and the statistical association between alleles $M$ and $X$ (described by gametic phase linkage disequilibrium, $D_{MX}$, which is the covariance between $M$ and $X$ alleles in sperm/pollen after sperm/pollen migration), weighted by the marginal frequency of the diploid genotype at the $\mathcal{F}$ locus in females/styles (i.e. before migration), $p_{i}$.

$p_{X^{''}}=p_{FF}\times\frac{p_{X}^{'}+c\left( D_{MX}^{'}+p_{x}^{'}p_{m}^{'} \right)}{1-cp_{m}^{'}}+p_{Ff}\times\frac{p_{X}^{'}+c\left( D_{MX}^{'}+p_{x}^{'}p_{m}^{'} \right)}{1-cp_{m}}+p_{ff}\times\frac{p_{X}^{'}}{1}$ (S4)

The change in frequency of an allele linked to $\mathcal{M}$ between pollination (mating) and fertilization is therefore

$\Delta p_{X\text{ fertilization}}=\left( 1-p_{ff} \right)\frac{c\times D_{MX}^{'}}{1-cp_{m}^{'}}$ (S5)

As such, the alleles in positive linkage disequilibrium with the sperm/pollen compatibility allele $M$ always increase in frequency during fertility selection because of the fertilization advantage, so long as there are some incompatible females/styles in the population. Because linkage disequilibrium between $M$ and $F$, as well as $M$ and $A$, is never less than zero, all teosinte alleles increase in frequency in both populations by fertility selection (Fig 3B).

#### The generation of trans linkage disequilibrium during fertilization.

We begin with two diverged populations, fixed for alternative alleles at local adaptation and sperm/pollen compatibility loci and with different allele frequencies at the female/stylar incompatibility. As such, LD between sperm/pollen compatibility and locally adaptive alleles is initially very large, and is broken down by migration and recombination.

This incompatibility system generates *trans* LD between sperm/pollen and female (stylar) loci — that is, compatibility rules generate a statistical association between paternally-derived $M$ alleles and maternally derived $F$ alleles. Before selection, this *trans* LD equals

$D_{MF,trans}=\frac{cp_{m}^{'}p_{M}^{'}p_{F}p_{ff}}{1-cp_{m}^{'}}$ (S6)

Clearly, this also indirectly generates *trans* LD between maternally-derived female incompatibilities and loci in LD with the sperm/pollen compatibility allele (or deviations from Hardy-Weinberg equilibrium at the $F$ locus). *Trans* LD is converted to *cis* LD by recombination.

### Local adaptation

Genotype frequencies at the local adaptation locus $\mathcal{A}$ after selection follow the standard population genetic equation $p_{i}^{'}=p_{i}w_{i}/\overline{w}$.

With multiplicative fitness effects, at a single local adaptation locus in the population in which $A$ is favored, genotypic fitnesses are: $w_{AA}=1$, $w_{Aa}=\left( 1-s \right)$, and $w_{aa}=\left( 1-s \right)^{2}$, and mean fitness equals $\overline{w}=1-2p_{a}s+s^{2}p_{aa}$. After selection, genotype frequencies are:

$\begin{matrix} p_{AA,\text{next gen}} & =\frac{p_{AA}^{‴}}{\overline{w}} \\ p_{Aa,\text{next gen}} & =\frac{p_{Aa}^{‴}\left( 1-s \right)}{\overline{w}} \\ p_{aa,\text{next gen}} & =\frac{p_{aAa}^{‴}\left( 1-s \right)^{2}}{\overline{w}} \end{matrix}$ (S7)

and the change in genotype frequencies between fertilization and viability selection are

$\begin{matrix} \Delta p_{AA,\text{next gen}} & =\frac{p_{AA}^{‴}-\overline{w}p_{AA}^{‴}}{\overline{w}}=\frac{sp_{AA}^{‴}\left( 2p_{a}^{‴}-sp_{aa}^{‴} \right)}{\overline{w}} \\ \Delta p_{Aa,\text{next gen}} & =\frac{p_{Aa}^{‴}\left( 1-s \right)-\overline{w}p_{Aa}}{\overline{w}}=-\frac{sp_{Aa}^{‴}\left( 1-2p_{a}^{‴}+sp_{aa}^{‴} \right)}{\overline{w}} \\ \Delta p_{aa,\text{next gen}} & =\frac{p_{aAa}^{‴}\left( 1-s \right)^{2}-\overline{w}p_{aa}^{‴}}{\overline{w}}=\frac{-2sp_{aa}^{‴}\left( 1-p_{a}^{‴}-s\left( 1-p_{aa}^{‴} \right)/2 \right)}{\overline{w}} \end{matrix}$ (S8)

.

With multiplicative fitness, the locally maladapted allele always decreases by direct selection, regardless of allele frequency or inbreeding coefficient (Fig S8).

#### Linked selection during local selection.

After fertilization, selection directly changes the frequency of the local adaptation loci as described above. This also changes the frequency of loci statistically associated with the locally adapted allele. The change in frequency of an allele, $X$ (e.g. $F$ or $M$), linked to $\mathcal{A}$ by linked selection is determined by the selection coefficient $s$ and zygotic LD, and equals:

$\begin{matrix} \Delta X= & \frac{s\left( 2D_{XX,A}+D_{Xx,A} \right)+s^{2}\left( D_{XX,aa}+D_{Xx,aa}/2 \right)}{1-2p_{a}s+s^{2}p_{aa}} \\ & \text{where} \\ D_{XX,A} & =p_{XX}p_{a}-p_{XXa} \\ D_{Xx,A} & =p_{Xx}p_{a}-p_{Xxa} \\ D_{XX,aa} & =p_{XXaa}-p_{XX}p_{aa} \\ D_{Xx,aa} & =p_{Xxaa}-p_{Xx}p_{aa} \end{matrix}$ (S9)
